# The Present Status of Dental Knowledge in the Medical Profession

**Published:** 1894-09

**Authors:** 


### THE PRESENT STATUS OF DENTAL KNOWLEDGE IN THE MEDICAL PROFESSION.

The teeth, sustaining as they do, intimate anatomical and physiological relations with some of the most important organs of the body, both by continuity of structure and by nervous and vascular communication, not infrequently are found to be the predisposing or exciting cause to some of the most formidable lesions known to pathology. In view of this fact, it is to be regretted that the almost daily practice, of many physicians, reveals a want of appreciation of the important pathological indications so frequently furnished by diseased conditions of these organs.

The two professions, dentistry and medicine, naturally meet and merge at certain points; and as Dr. Kirk has said in an editorial in the *Dental Cosmos*, "the difference in the mental attitude of the physician and dentist towards problems which they meet in common and which cannot be wholly relegated to the sphere of either, is important when regarded from the stand-point of its results." The same writer, some time since, gave a critical review of a work on "The Diseases of the Mouth in Children," by F. Forchheimer, M.D., Professor of Physiology and Clinical Diseases of Children, Medical College of Ohio. This brought out several expressions of opinion from a number of writers in various periodicals, both medical and dental.

Dr. Forchheimer's book, which received favorable notices and commendations by the medical journals, condemns the use of the gum lancet as a therapeutic measure, on the following grounds:

"1. It is useless: *a*, as far as giving relief to symptoms; *b*, as far as facilitating or hastening teething.

"2. It is useful only as blood-letting, and ought not to be used as such.

"3. It is harmful: *a*, in producing local trouble; *b*, in producing general disturbances, on account of hemorrhage; *c*, in having established a method which is too general to do specific good and too specific for universal use.

"4. It is to be used only as a surgical procedure to give relief to surgical accidents."

This all shows a lamentable, an inexcusable, want of appreciation of the many important pathological indications so frequently accompanying the advancement of the deciduous teeth. The attitude of the medical profession in this matter is further set forth in the following extract from an editorial in the *Journal of the American Medical Association*:

"We think it can be said, without fear of contradiction, that there is not a single positive observation which has ever been recorded to prove that dentition produces general or reflex symptoms. It is undeniable that, at the period in life when dentition is in progress, the infant is subject to certain disorders which occur much more commonly than at any other period of life. If it could be shown that dentition was the only peculiarity of the infant, then its causative influence would be clear. But dentition is not the only peculiarity of the infant, and coexisting phenomena can only be classed as *coincident*." A remarkable statement coming from one who is supposed to be a teacher, and whose writings are expected to have a certain influence in his profession. It is true that all the diseases of infancy cannot be ascribed to dentition (nor do we attempt to), but there are certain well-marked conditions which are produced by reflex action, and which are unmistakable to the trained intelligence. There is considerable literature upon the subject to which we could refer; the most notable is the very able and exhaustive paper by Professor A. P. Brubaker, in the third volume of the "American System of Dentistry." Attention is called to the following quotation: "It is a reasonable supposition that the laws of nerve-force correspond to the same general laws as other forces. As no force is ever lost, but must, when it disappears, appear as some other mode of motion, so peripheral irritation—continually travelling inward—is either reflected over and outward by other routes to other organs, or is stored up in interrelated centres until morbid function results in them."

We have not the space to quote fully from authorities, but the following from the pen of Professor Truman—in writing editorially in this journal—seems to apply admirably to the point of "coincident:" "It is certainly too late in the progress of medical science," he says, "to assert that no reflex disturbance occurs in teething, or that it is solely dependent upon the eruption through the gum-tissue. Such a statement seems to indicate a blind perversion of facts, and which could never be made truthfully by one who had watched the progressive phenomena resulting from dentition and the relief afforded by the use of the lance."

As further evidence of the need of a higher standard of dental knowledge for medical practitioners, especially those who incidentally figure as teachers, we cite the following: A well-known surgeon, who is also an author and a professor, cautions his students to "be sure, before extracting a tooth, to cut the *ligamentum dentis*." He then relates that its discoverer "first detected it *by means of a microscope*, in the maxilla of a hog, and afterwards demonstrated it in the human subject." Another interesting instance is given by a contributor to one of our journals. He was requested by a practising physician, who was also an editor of a medical journal, to see a child who was "suffering from some trouble which was difficult to diagnose,—a most extraordinary and distressing case. A portion of the superior maxillary bone was denuded; integument sloughing away, etc." Upon examination, there was found protruding some distance above the gum margin the denuded root of a decayed temporary tooth, such as we frequently see; this being the sole cause for the alarm. We frequently learn of physicians allowing patients to suffer from alveolar abscess, putting them to bed with the information that they are suffering from "neuralgia." And quite recently a lady called upon us with the request to have a small amalgam filling removed. It was the only alloy filling she had and was in good condition. She had been sent by her physician, who claimed he could not cure a throat affection, from which she was suffering, as long as the filling was retained.

Our literature is replete with authentic reports of such cases, but an excellent illustration of the point in hand, one that has never been published, we take from the practice of Professor Peirce. A well-known neurologist had under his care a lad suffering from chorea. The case was presented to Dr. Peirce, who, on examination, found several persistent deciduous teeth interfering with the normal eruption of the bicuspids, and these at his suggestion were removed, and within a few days the distressing symptoms disappeared. Under the care of the same was a girl subject to attacks of epilepsy, which were thought by the attending physician to be incurable, but upon the removal of several deciduous teeth and two diseased first molars, the marked improvement was evidence conclusive that dental irritation was in this case also an important factor.

A well-known dental writer, the late Professor Richardson, has truly said that it is the reproach of *curative* medical science that patients are daily drugged for the relief of maladies which, being purely symptomatic of, or dependent on, dental diseases or irritation, are diagnosed and obstinately and perseveringly treated as idiopathic affections.

It is also a reproach of *conservative* medical science that countless numbers of teeth are, year after year, uselessly and mercilessly sacrificed through the direct agency, or complicity, of many medical practitioners; and a reproach of *sanitary* medical science that, while the greatest circumspection is displayed in guarding the many approaches to the citadel of health, one of the most exposed and vulnerable points, whose defence is essential to the general safety, should be left so entirely uncared for.

Of course, all medical practitioners are not thus perverse and stupid. We number among our clients several of our most active physicians, who are always anxious, through personal and professional intercourse, to secure any information of value. Many of us, too, are called in consultation by physicians who recognize the close relationship of dentistry and medicine. But these are the exceptions—they are the salt of the profession. It is the average medical practitioner (which is the majority) to whom we allude.

There are obvious reasons, too, why the general practitioner can not be said to be entirely at fault where the teeth are concerned. His partial vindication is in the fact that the present instrumentalities employed as means of imparting a medical education afford little or no opportunity for the acquirement of the truths so amply revealed by observation and experience to our own profession.

It is therefore desirable—and it would be a wise step in the higher education of medical students—that a chair of dental pathology be established in our medical schools, and that both student and practitioner should keep in touch with our best current literature. G. W. W.

